# Space Research Ethics

**DOI:** 10.1007/s11948-025-00576-7

**Published:** 2026-01-07

**Authors:** Oskari Sivula, Mikko Puumala, Minna Palmroth

**Affiliations:** 1 Department of Philosophy, Contemporary History and Political Science, University of Turku, Assistentinkatu 7, Turku, 20014 Finland; 2 Department of Physics, University of Helsinki, Helsinki, 000014 Finland

**Keywords:** Research ethics, Space science, Space ethics, Sustainability, Ethical review, Space governance

## Abstract

In this paper, we characterise the field of space research ethics and the moral and political landscape it operates on. To lay the groundwork for our ethical analysis, we begin by introducing the diverse ways space research is conducted in practice and by discussing ongoing changes in its operational environment. We note that space research is necessary to facilitate our modern way of life and is integral to green and digital transformation. Following that, we identify key space research ethical issues in different domains and suggest tentative elements that trigger the need for research to undergo ethical review, to forward discussion and debate. The difficulty is to find suitable guidelines that are defined clearly enough to be action-guiding but simultaneously broad enough to address the complexity and global nature of space research. In the long term, the most feasible approach may be to define a tailored set of research ethical guidelines for particular fields, particular space environments, and particular research goals. We further address how the collective and global nature of space activities adds additional layers of challenges regarding the research ethical responsibility of the scientific community. In this context, we suggest that research ethics is not only a set of specific moral codes developed as a professional ethic for researchers, but also an institutionalised way to guide and support research activities and the integrity of researchers to maintain trust within the research community and the society at large.

## Introduction

In recent years, we have seen substantial growth in space activities, anticipating that space research activities are also likely to increase and take new forms in the future. Yet, there has not been a systematic discussion about research ethics for space research. The need for such a discussion, however, has been noted. For instance, in an editorial note for *Research Ethics* Hunter (2013, p. 150) asks whether experiments like the envisioned Mars One “ought to be under the purview of research ethics committees”, Rahimzadeh et al. (2023) proposes an ethical framework for human research in commercial spaceflight, Marino et al. (2023) discusses ethical considerations for analogue fieldwork in astrobiology and related research, and Haramia et al. (2025) argue for a more expansive understanding of what can contribute to value to guide space research and exploration. Many space activities, including research, have guidelines (see e.g. Barbier, 2018) and regulations, but an overarching, detailed review is still lacking. In Finland, there is an attempt to develop national guidelines (project LYTE)^1^ that address the environmental impacts of research, the space environment, as well as Earth’s atmosphere, being part of the guidelines. In November 2024, the authors of this paper were invited to give an introduction to potential ethical issues regarding space research, and this paper stems from that discussion.

The emerging field of space ethics can be seen as a response to the growth in space activities. In some sense, many space ethical topics and issues, such as planetary protection, are simultaneously research ethical questions, but the discussion would benefit from taking a clear perspective of research ethics on the topic. That is, taking research ethics not only as a set of specific moral codes developed as a professional ethic for researchers, but also research ethics as an institutionalised way to guide and support research activities and the integrity of researchers to maintain trust within the research community and the society at large.^2^

While space research is to a large extent covered by existing research ethics on Earth, it is still worthwhile to appreciate the particular research ethical concerns of space research to advance responsible conduct and to anticipate ethical challenges in this growing field. What makes the task difficult is that space research is often varied, highly multidisciplinary, carried out in cooperation with different international parties from various backgrounds, sometimes extremely expensive, and operates in a context of different national space legislations, vague international treaties, and various levels of other existing regulations. The difficulty is to find suitable guidelines that are defined clearly enough to be action-guiding but simultaneously broad enough to address the complexity and global nature of space research. In the long term, the most feasible approach may be to define a tailored set of research ethical guidelines for particular fields, particular space environments, and particular research goals. The purpose is not to make space research more difficult or unnecessarily restrict it, but in the best scenario, make it easier to navigate a complex set of problems in cooperation with a wide array of parties.

In general, different space activities bring about immense benefits (see, e.g. Palmroth, 2024). Space research brings answers to urgent questions that help us understand the world and monitor global problems like climate change. Hence, a balanced approach in considering ethical guidelines is needed. Space research does not operate in a vacuum, but is part of a complex system of operations aimed at improving life on Earth. What is the role of the research community in this context, what are its responsibilities, and how does space research contribute to various adverse effects? We do not argue that an entirely new research ethics for space is needed, since much of it is already covered by existing guidelines and national or regional legislation and regulations. Rather, we wish to highlight the distinctive nature of space research and call for greater consideration and discussion of its ethical concerns.

To forward discussion on the subject, this paper characterises the field of space research ethics and the moral and political landscape it operates on. We begin by introducing the diverse ways space research is conducted in practice and discuss how its operational environment is changing. Following that, we identify key space research ethical issues in different domains and suggest elements in research that call for ethical review. Finally, we discuss how the collective and global nature of space activities adds additional layers of challenges regarding the research ethical responsibility of the scientific community.

## Space Research in Practice

The different research fields under the umbrella of ‘space research’ are perhaps most comprehensively listed by the International Committee on Space Research (COSPAR) Scientific Commissions: (A) Space Studies of the Earth’s Surface, Meteorology and Climate; (B) Space Studies of the Earth-Moon System, Planets, and Small Bodies of the Solar System; (C) Space Studies of the Upper Atmospheres of the Earth and Planets Including Reference Atmospheres; (D) Space Plasmas in the Solar System, Including Planetary Magnetospheres; (E) Research in Astrophysics from Space; (F) Life Sciences as Related to Space; (G) Materials Sciences in Space; (H) Fundamental Physics in Space (COSPAR Committee on Space Research, 2025). All these are experimental fields with observations in their core, and hence require space-based infrastructure, even though Commissions A, C, and D especially involve an important ground-based component complementing space operations. Space research started in 1958 with the first satellite that carried a scientific payload, Explorer-1, and resulted in the discovery of the radiation belts surrounding the Earth (Van Allen, 1959). Since then, numerous discoveries have been made, some of which have later been adopted in industries and services, indicating that space research is an integral part of modern society. However, satellite observations involve designing, building, testing, launching, calibrating, and operating, all of which cost money and use natural resources. Ground-based complementing space research also requires similar operations, save for the launches. In addition, modern space research would not be complete without numerical models, some of which require large supercomputers and data centres. Since sustainable computing is a trend, it is desirable that computing requiring a large amount of electricity is carried out in carbon-negative computing centres, like the CSC data centre in Kajaani, Finland.

According to the European Space Agency (ESA), recent years have seen an exponential increase in the use of space (European Space Agency, 2024). During the Cold War, approximately 150 satellites were launched annually, largely motivated by military applications. Around the midway between 2010 and 2020, the launch market faced a complete paradigm change when commercial launches started to be available. Before this, only large space actors like the ESA, USA, Russia, China, and India were able to launch. Commercial space launches opened commercial market possibilities in space. In 2024, over 2500 satellites were launched, and many more are planned for the future (see, e.g. Muelhaupt et al., 2019). The expanding number of launches has drawn interest to investigate the environmental impact of space operations. For example Dallas et al. (2020) review the topic and conclude that rocket launches are involved with stratospheric ozone depletion, mesospheric cloud formation, climate change, and ecosystem and human toxicity. It is to be emphasised that, for example, in 2024, less than 1% of all satellite launches were motivated by science, and the vast majority of the environmental impacts are hence caused by the commercial use of space.^3^

By Newton’s laws, a physical body keeps moving with a constant speed if no force acts to decelerate or accelerate it. In space, the only forces available are the friction provided by the expanding atmosphere that is still acting at around 500 km altitude, and the propellant within the satellite. This means that satellites above around 500 km altitude will stay in their orbits if they are not actively manoeuvred or removed (or *de-orbited*). A resulting outcome has been the growing number of space debris. Large space organisations convened an Inter-Agency Space Debris Coordination Committee that has agreed upon a guideline that satellites should be removed from their orbits after 25 years of the mission’s end (Inter-Agency Space Debris Coordination Committee, 2007), while the USA has a new 5-year rule.^4^ On Low Earth Orbits, de-orbiting is typically carried out either by letting the satellite decelerate towards the atmosphere, where it burns or, if the satellite is large enough, aiming it to the Pacific Ocean by burning fuel at the end of its mission. On higher orbits like the geostationary orbit, decommissioning is carried out by moving the satellite to a so-called graveyard orbit, in which case the satellite’s slot is freed for the next mission. It is clear that if satellites are left in orbit at the end of a mission, they will contribute to the existing debris. This increases the risk of a so-called Kessler syndrome (Kessler & Cour-Palais, 1978), which means that debris objects start to collide exponentially, which catastrophically increases the number of debris, leading to a situation in which orbits would become unusable.

It is to be emphasised that one of the largest factors contributing to satellite loss on orbit are the physical conditions in space, collectively termed as space weather (Doherty et al., 2004), which largely belongs under COSPAR Commission D. This is an active research field which is not understood at the level that it could be used to protect the space assets from turning into debris. Finnish Centre of Excellence in Research of Sustainable Space (2018–2025)^5^ was the first consortium in the world to comprehensively investigate space weather as part of the sustainable use of space, with the aim of preventing the formation of new debris from new satellite launches. This includes understanding the highly unpredictable and dynamic space weather, especially space radiation, and developing cost-efficient de-orbiting technologies that could de-orbit satellites without fuel, using the forces that are available due to the plasma environment in space. Hence, it is important to understand that especially space physics, space weather and technology research, which necessarily require space operations, are also mandatory to understand the conditions that create and remove debris (Palmroth et al., 2021). This dichotomy was outlined in a recent article (Palmroth & Hukkinen, 2025), discussing the two branches of sustainability theory: Should actions be prevented altogether for the sake of environmental sustainability, or should new methods and processes be developed in order to maintain a balance between a sustainable environment and, for example, commercial operations? It is essential to understand that space weather research is also required to understand the reliability of the terrestrial critical infrastructure, and hence the discussion is not only concerning space but the modern way of life, critically dependent on space-based operations (see Olla, 2009). This indicates that space operations are required to understand how to act sustainably in space, and, for example, what the sustainability implications of space weather for modern society are.

## Space Research Domains of Ethical Interest

One of the goals of research ethics is to anticipate and minimise the harms created by research. This means that we need to have a clear picture of what the potential impacts of research are.^6^ Here, we have listed the most salient ethically relevant areas of interest related to space research.

### Human and Non-Human Animal Research Subjects

In space research, different lifeforms are sometimes sent into space, most notably to the International Space Station. Animals,^7^ plants or fungi are launched beyond Earth to study them, or humans fly to space to conduct research there. Space stations have served as unique laboratories to study human health in space and the physiology of animals and other living organisms. These questions are becoming more urgent due to the United States’ plans to send a crewed mission to Mars within a tight timeline. It should be noted, however, that most space professionals deem such a timeline unrealistic (Maiwald et al., 2024; Verseux et al., 2025), and the US ambitions regarding Mars seem to be readily changing.

Because the space environment is inherently dangerous, ethical scrutiny is warranted when sentient beings are sent to space. Therefore, this is a clear research ethical element that calls for ethical review. A corresponding guideline could be:

#### G1:

*Ethical review is needed when the research involves sending humans or non-human animals to space.*

Human and animal research is governed by national or regional regulations and guidelines.^8^ In this sense, G1 reiterates the obvious. Nevertheless, for the sake of comprehensiveness, it is worth bringing up here. It is also worth recalling that research ethics committees “help researchers catch themselves when they might be tempted to put the ends of science above the interests of their research participants” (Hunter, 2013, p. 151).

Regarding human and animal subjects in space research, existing regulations and policies – such as the *COSPAR Policy and Guidelines for the Utilization and Care of Animals Used in Space Research* (Stabekis, 2007) – as well as well-established principles and recommendations, can provide guidance for the ethical review, since they represent cases that are extensively discussed in the bioethical literature. Still, there is the worry that “many research ethics committees will not have the necessary expertise to conduct quality, comprehensive reviews of spaceflight research” (Rahimzadeh et al., 2023, p. 1411). Thus, experts on spaceflight and space research should be consulted for this and other elements highlighted through Section “Space research domains of ethical interest”.

The issue of human and animal use in space research may become increasingly important when the costs of launches continue to decrease. Falling launch costs may result in human space flights becoming more common, and also entail a growing number of animals being used in biomedical space research (cf. Pozzebon, 2024).^9^ As Rahimzadeh et al. (Rahimzadeh et al., 2023, p. 1409–1409) note, “[n]ow is the opportune time to develop clear rules for ethical [commercial spaceflight participants] research while space activities are ramping up and the regulatory environment for commercial spaceflight is actively being shaped.” However, one should bear in mind that the boundary between commercial research and public research may not be clear-cut; ideally, both would be covered by a single framework. Indeed, it seems problematic if research is overly regulated compared to commercial activities in space.

A related area of interest is space analogue missions where analogue astronauts go to different environments to, for example, test equipment, study behavioural stressors, and learn about team dynamics. These often include long stays in isolated and extreme environments that share or imitate features of the studied target environments or populations. This means that also for such studies, research ethical review is called for to make sure that enough attention is paid to issues such as informed consent, safety (see Klicker et al., 2023), and proper consideration for the local environment and community (see Marino et al., 2023). Here, again, existing guidelines and principles for research with human participants can guide the research ethical review.

### Environmental Impact on and Off-Earth

Interestingly, in space research, we need to consider living organisms even when we are not explicitly launching life into space, but also when we are ‘only’ sending spacecraft and science equipment. This is because microorganisms may travel with these objects to other celestial bodies, something known as *forward contamination*. On Earth, in most cases, that is, unless research is dealing with isolated environments or groups, one does not have to worry about microbes piggybacking on researchers, vehicles, or instruments because microbes are everywhere. The same is not true for space environments. This issue is well-acknowledged in the scientific community, and forward contamination is especially important for scientists since contamination may significantly hamper scientific investigations. That is why COSPAR maintains a policy on planetary protection (Ehrenfreund et al., 2024). Regarding Mars, these plans, of course, become obsolete in case someone succeeds in sending a crewed mission to Mars in the coming years, because the minute an astronaut walks on the Red Planet, contamination is evident.

What should be noted is that COSPAR is not an overall ethical authority in the sense that their evaluation is done mainly based on the protection of the value of astrobiology research (Cockell, 2005; Persson et al., 2018; Puumala et al., 2025). So, it does not entail a full-fledged ethical evaluation. This means that space research should comply with planetary protection protocols, but that is only the minimum. Sometimes, planetary protection considerations should be accompanied by a wider ethical evaluation. This could be the case, for example, if a celestial body is proposed to be intentionally seeded for a “biosphere genesis experiment” (see Cockell et al., 2025) or if in the future living microorganisms will be studied in the interstellar space environment as suggested by Lantin et al. (2022) since such studies could entail a risk of seeding exoplanets with life.^10^

COSPAR’s policy addresses not only forward contamination but also *backward contamination.* The idea behind it is that we ought to protect Earth’s life from the harmful effects of potential extraterrestrial life that may come to Earth via sample return, for example. In light of this, we can identify a second space research ethical area of interest.

#### G2:

*Ethical review is needed when the research creates a non-negligible risk of forward or backward contamination.* 11

In addition to the ethical questions related to living organisms in space, space research may have other environmental impacts that are ethically relevant. For example, all human activity – space research included – creates greenhouse gases.^12^ Moreover, as outlined in Section “Space research in practice”, rocket launches are involved with impacts that may be detrimental to the well-being of humans and other living organisms. However, perhaps the most pressing problem of this sort is the increasing amount of debris surrounding Earth. While the portion of research missions of all current Earth-orbiting missions is tiny, it is important to consider new ways to carry out space research to maintain sustainable conditions for the long-term usability of space. There are no binding international space laws, and the only binding regulation is found from national legislations of the countries involved, referred to as “launching states,”^13^ and these laws vary from country to country (Palmroth et al., 2021). For example, in Finland, the space law^14^ stipulates that launching states are liable to ensure that the launch and operation of the mission will not harm others in orbit.

Launching a large satellite for research purposes could create a significant physical impact if it creates space debris that makes the Kessler syndrome more likely. In practice, one can only require that national space law stipulations are met. Another question entirely is multinational organisations. An example of this is ESA’s Envisat mission, which aimed to study Earth’s atmosphere and climate change. After losing communication, Envisat became a significant debris hazard. ESA holds responsibility for minimising collision risks, but safely removing Envisat from orbit would require substantial advancements in space technology, including a very demanding successful docking.

It should be highlighted that it is not only in Earth’s orbits that research may have significant environmental impacts, but also elsewhere in outer space. Space activities with environmental impacts beyond Earth and its orbits can range from already tested asteroid deflection operations (DART-mission) to the more speculative enterprise of planetary-scale engineering that some have proposed.^15^

A unique issue about outer space environments is that in space, we can have environments that are truly natural or pristine, that is, areas with no human influence. Such wilderness is ethically important according to many philosophers (see, e.g. Elliot, 1982; Katz, 2012; Siipi, 2004), so we may need to pay attention to this.^16^ A further distinctive issue is that many space environments behave very differently compared to environmental systems on Earth. For example, any impact left on the Moon will stay there for millions of years because erosion is considerably slower. After all, the Moon lacks an atmosphere, tectonic activity, and biological systems, meaning that it is a much less dynamic system than Earth. Research activities in these environments can be environmentally harmful in the sense that they may result in losing environmental or aesthetic value.^17^ Determining the nature of this value requires further inquiry, but it should be noted that even on Earth, some geologically interesting environments are also deemed valuable in their own regard and have merited protection. Thus, a third tentative guideline could be the following:

#### G3:

*Ethical review is needed when space research entails significant harmful environmental impacts on Earth or space environments.*

A clearer picture of what constitutes a significant impact is something that needs to be clarified. Clearly, not all impacts are significant enough to merit ethical evaluation. Yet at some point, impacts can shift from being trivial to being considerable enough to warrant ethical scrutiny. Where exactly this line is in a given situation is difficult to determine. Also, there is the second-order consideration of how we ought to act under such uncertainty. Perhaps we should adopt a precautionary attitude in certain cases to accommodate for such uncertainty, as the maxim – *in dubio pro natura* – suggests (see, e.g. Ahteensuu, 2007). However, the philosophical complexities of this are too big a subject to be settled here and hence must be left for future research.

### Cultural Heritage

In recent years, there has been a growing interest in ‘space heritage’, which refers to the material culture left behind from space exploration. This includes features such as “footprints and trails created at Apollo 11’s Tranquility Base on the Moon in 1969, the first impact on the lunar surface by the USSR’s Luna 2 in 1959” (Holcomb et al., 2024, p. 1490), and “the USSR’s Mars 2 lander and PrOP-M rover, […], the American Viking 1 lander, which was the first lander to operate successfully on Mars, and Ingenuity, which was the first autonomous helicopter to fly on Mars.” (Ibid.) In 2025, this enthusiasm led to sites on the Moon being added to a list of threatened cultural sites upheld by the World Monument Fund (2025). A similar ethos of the need to protect space heritage can be found in the US national legislation.^18^ Because of this collective interest in preserving space heritage, a further element that requires ethical evaluation can be identified. A corresponding guideline could be:

#### G4:

*Ethical review is needed when space research entails a significant risk of permanent destruction of space heritage.*

While such a guideline for space research ethics seems sensible, it must be emphasised that there are still unresolved issues regarding the demarcation between heritage and simply litter, which may be easily politicised. This is an issue we will not attempt to resolve here.

### Dual-Use and Signaling

Finally, we want to consider the dual use of science instruments and the transmission of detectable signals to outer space as relevant ethical considerations for space research. The most prominent example of dual-use related to space studies is satellites because they may become kinetic weapons if they have manoeuvring capability. The risk of dual-use is an element that calls for attention, and a related guideline could be:

#### G5:

*Ethical review is needed when space research entails a significant risk of misconduct via dual use.* 19

Furthermore, we want to bring up another less-discussed case that combines both features of dual-use and messaging to the stars, namely, high-power transmit radars. Such science instruments can be used for something other than what they were initially designed to do. For example, such scientific facilities have been used to send a Doritos advertisement towards a star 42 light-years away from Earth in the Ursa Majoris constellation by the Pepsi company.^20^ There have also been more serious attempts to make contact with potential extraterrestrial intelligence. For instance, in 2018, METI International, together with a festival called SONAR and the Catalonia Institute of Space Studies, sent messages to a star only 12,4 light-years from Earth that has a potentially habitable exoplanet.^21^

We are not taking any stance on these experiments but simply acknowledge that they are in an ethically grey area.^22^ This means that there should be some ethical oversight about the use of space science equipment for something so different from what they were designed for, and that the possible responsibility lies partly also with the parties renting the instruments.

Because METI-international and their colleagues claim that what they are doing is a legitimate scientific test – ‘let us send a signal and see if anyone responds!’ – a sixth guideline to trigger ethical evaluation could be the following:

#### G6:

*Ethical review is needed when space research entails transmitting easily detectable signals to possibly habitable areas in outer space.*

Having examined key elements of space research that demand ethical oversight, we now turn to its collective nature and the ethical questions that arise from it.

## Collective Research Ethical Problems in Space Research

Space researchers do not operate in isolation from the rest of society. The orbits and the different environments on celestial bodies like the Moon or Mars are also points of interest for many different actors. Moreover, the expenses and the requirement of specific expertise, equipment, and technological capabilities often mean that it is conducted in a global context. It occurs in a legal framework that is not only national but also international. It also observes phenomena that are not usually confined to some particular country, and its impacts are sometimes global.

Such circumstances invite conflict, strengthened by the fact that resources and accessible environments in *near-term technologically accessible space* are limited and thus are also contestable. We operate in circumstances of scarcity, and scientific value is only one of many different types of value people are looking for in space. Earth’s orbits are a paradigm example of a scarce resource – we can oversaturate them with too many satellites. Thus, they are a resource the research community needs to share among themselves, but also with other space actors.

According to one prominent interpretation of current international agreements, space should be treated as a commons.^23^ It is a common-pool resource, which belongs to all humankind. The 1967 Outer Space Treaty states that outer space is the province of all mankind, and highlights the principles of free use and exploration. This also creates challenges, similar to most common-pool resources: the tragedy of the commons (Morin & Richard, 2021). This collective action problem arises when there is a scarce common-pool resource that can be overused to the detriment of all related parties. To paraphrase the classic example: for each research party, it is in their interest to increase their research activities in the commons. If each research party increases their activities, the commons will be saturated and all parties will lose. Increasing the number of satellites in Earth’s orbits resembles the structure of this kind of collective action problem, and research is not the only aim for using the orbits. How to manage the use and distribution of the orbits, and assess the priority of different aims? A standard approach to the tragedy of the commons problem is to increase cooperation among different parties. Increased research cooperation and also coordination between parties with different aims is important. For instance, it is standard practice that good scientific conduct includes sharing data, and the tragedy of the commons highlights the need for this in space research.

As noted in Section “Space research in practice”, only a small proportion of the uses of Earth’s orbits are scientifically motivated. Moreover, if scientific uses of common-pool resources generate more value than other, more frivolous reasons, these contributions to collective action problems are more easily justifiable (see Schwartz, 2020). Yet, it should be acknowledged that scientific research can nevertheless contribute to these problems, even if to a lesser degree.

Responding to the collective action problem gets even more complicated when the internationality and global impacts of space research are considered. This has research ethical ramifications. One important research ethical problem is the so-called *ethics dumping*, which refers to the ethically questionable practice of conducting research in areas where there are less strict research ethical rules and guidelines. A related worry is that noncompliance and free-riding can skew the entire practice. For instance, if some country has looser regulations or lower research ethical standards and it would not commit to international treaties, regulations, or guidelines, it could harbour promises of a fast-track for scientific progress.^24^ Science is a competitive endeavour, so this could make it tempting to lower the standards to keep up the pace. What are the incentives to maintain high research ethical standards, if, for example, limiting national research with national guidelines has no tangible effect on worldwide practices and outcomes? And even if high research ethical standards are maintained, how should the rest of the scientific community treat the data gathered with less ethically sound means? This highlights the need for shared standards and international cooperation.^25^

Another global question is related to environmental justice (Klinger, 2019). The benefits and harmful effects of space research activities are not always distributed fairly. Light pollution, potential atmospheric effects of de-orbiting satellites (Flamm et al., 2024), and space debris can cause damage, so it is plausible that, at the very least, the benefits should also be spread, calling for open science practices. Also, some research facilities are located in Indigenous territories or lower-income countries, raising further issues of justice (Milligan, 2023).

Related to the latter point, it may be questioned whether space research, as it currently stands, is diverse and inclusive enough. As Aganaba et al. (2025) have noted:

[M]any low- and middle-income countries (LMICs), Indigenous Nations and communities and small states are missing from debates around space activities. Their concerns around how people operate in space, the environmental consequences, Indigenous rights, diverse views of cosmology and demands for political and scientific participation are typically dismissed.

In light of this and the fact that OST states that outer space is the province of all humankind, space researchers should pay attention to the inclusivity and equity of their practices. Strengthening international collaboration and incorporating diverse worldviews about space can benefit science. At the very least, scientific research should always be conducted in a way that respects all groups and their rights.

These considerations set the stage for scientific research in the space environment, and they should also be a key factor for determining ethical guidelines for space research. For instance, when new research projects compete over scarce resources and contribute to their overuse, they require a solid basis for justification. At face value, scientific goals seem more easily justifiable than other (e.g., economic) goals for the use of space resources (Schwartz, 2020). However, research ethics points out that such claims cannot go unqualified. The suggested guidelines in this paper show when further ethical assessment may be required. But there is also a broader question of the research ethical responsibility of the scientific community. What role should and can the space research community have in this highly global context? What are the roles and responsibilities of individual researchers, research groups, and research institutions in a broader context of international cooperation and research practices? There is no easy answer to these questions, and these considerations may not yield a separate research ethical guideline, but more of a broader task for the scientific community to set high standards, take leadership, and show example in the space exploration community. This calls for increasing (self-)awareness in the scientific community of this important role.

## Conclusion

While many space activities are crucial for the modern way of life, and space research can be seen as an especially valuable space activity, it does not mean that they are outside the realm of ethical deliberation. Instead, moral philosophy is needed to guide and inform space activities, research included. Thus far, the space ethics literature has lacked an explicit research ethical discussion about space research in general, and this paper has aimed to fill this gap. When approaching the topic of research ethics in the context of space, two further questions require careful consideration: first, how do space research activities link to other research ethical guidelines and the body of research ethical literature, and what can be learned from there? Second, special attention should be paid to what is unique in the context of space that merits separate inquiry. In this paper, we have merely touched upon these issues and hoped to spark further discussion.

The discussion was broad, aiming to serve as a launch for a more fine-grained discussion. We presented many examples of ethical issues pertinent to space research specifically and suggested a handful of tentative guidelines for space research ethics. Such guidelines are important and valuable, but, as we have noted, space research takes place in a global and complex setting where various and multifaceted ethical questions emerge. Thus, arguably, in addition to some set of guidelines, the science community needs to foster a spirit of responsible research ethics that is quite holistically understood. This includes using voices as scientists in matters of great importance, maintaining an international dialogue on research ethical issues specific to different fields, and educating themselves and consulting experts about the ethical conundrums that may concern research activities. By doing so, the community can better maintain public trust in space science.

## Data Availability

Data sharing is not applicable since we do not analyse or generate any datasets, as our work proceeds within a theoretical approach.

## Footnotes

1 For more information about LYTE, see: https://tenk.fi/en/projects/lyte-project.

2 Of course, the purpose of research ethics is to guide researchers in conducting their research in an ethically sound way, which hopefully strengthens public trust in science. Ethical conduct is the goal here, not trust itself.

3 In addition to the scientific and commercial uses of space, there are, of course, military and civil government satellites. Even accounting for these, most spacecraft launched are commercial satellites.

4 See, https://www.fcc.gov/document/fcc-adopts-new-5-year-rule-deorbiting-satellites-0.

5 See, https://www.helsinki.fi/en/researchgroups/finnish-centre-of-excellence-in-research-of-sustainable-space.

6 Or, as has been recently advocated for, what the *cosmic footprint* of our space activities are (see Normier et al., 2025).

7 Johnson (2015) has discussed the vulnerability of nonhuman animals who have participated in space research and argues that in many cases harms inflicted on ‘animal astronauts’ are not warranted because the justifications for space research are not strong enough.

8 In the European Union (EU), for example, animal research is governed by the EU directive “on the protection of animals used for scientific purposes” (Directive 2010/63/EU).

9 On the bioethical implications of commercial human spaceflight, see Langston (2015). For bioethics of possible future long-duration space missions see e.g., Szocik et al. (2020); Szocik (2021, 2023). The two issues that Konrad Szocik raises as distinctive for such missions from bioethical perspective are human enhancement and reproduction in space. If such missions are scientific in nature, these concerns could fall within the scope of space research ethics in the future. However, for now and in the short run, science missions are unlikely to involve reproduction in space or controversial forms of human enhancement.

10 On the ethics of planetary seeding, or directed panspermia, see e.g., Mautner (2009); O’Brien (2022); Sivula (2022, 2025); Soryl and Sandberg (2025).

11 What non-negligible specifically means in this context is an important question that deserves further inquiry and discussion. What can be said, though, is that, if the potential consequences of a materialised risk are catastrophic, then arguably even very small risks ought to be taken into consideration.

12 On the carbon footprint of Astronomy, see Knödlseder et al. (2022).

13 The Outer Space Treaty (Article VII) states that, Each State Party to the Treaty that launches or procures the launching of an object into outer space, including the Moon and other celestial bodies, and each State Party from whose territory or facility an object is launched, is internationally liable for damage to another State Party to the Treaty or to its natural or juridical persons by such object or its component parts on the Earth, in air space or in outer space, including the Moon and other celestial bodies. https://www.unoosa.org/oosa/en/ourwork/spacelaw/treaties/outerspacetreaty.html

14 See https://www.finlex.fi/en/legislation/collection/2018/63.

15 For a critical overview of terraforming Mars, see Forget et al. (2025). For a more optimistic view, see DeBenedictis et al. (2025).

16 For debates concerning naturalness and wilderness in the context of space, see, e.g., Lee (1994); A. R. Johnson (2020); Sivula (2024); Lindquist (2024).

17 For a related discussion, see, e.g., Rolston III (1986); Cockell and Horneck (2006); Matthews and McMahon (2018); Haramia et al. (2025).

18 See, https://www.congress.gov/bill/116th-congress/senate-bill/1694.

19 As one of the reviewers pointed out to us, this might be a guideline that will not be adopted easily, considering that dual use has since the beginning of the space era been an important driver of space exploration (see, e.g. Bowen, 2022).

20 Zaitsev (2012) describes the Doritos message as ‘Pseudo-METI’ or ‘stunt’.

21 See: https://www.sonarcalling.com/en/.

22 On the ethics of messaging extraterrestrial intelligence, see e.g., Vakoch (2016); Haramia and DeMarines (2019); Smith (2020); Haramia (2024). See also Peter Hatfield & Leah Trueblood (2022) who discuss democratic approaches to SETI and METI to make decision about them more legitimate.

23 This is a debated interpretation, but it goes to show that the legal context of space activities is controversial and unclear, which adds to the complexities of space research ethics. For discussion, see Tepper (2019); Weeden and Chow (2012).

24 However, on the contrary, some have argued that it is high research ethical standards that lead to better quality science. For a recent paper defending this view, see Miteu (2024).

25 There are many ways the scientific community can be its own gatekeeper for better practices. One of them is academic journals and the requirements they have for publishable manuscripts. Thanks to the anonymous reviewer for suggesting this idea.
